# A Pilot study of the Sharing Risk Information Tool (ShaRIT) for Families with Hereditary Breast and Ovarian Cancer Syndrome

**DOI:** 10.1186/1897-4287-10-4

**Published:** 2012-04-12

**Authors:** Ani Kardashian, Julia Fehniger, Jennifer Creasman, Eleanor Cheung, Mary Stanley Beattie

**Affiliations:** 1 University of California San Francisco School of Medicine, 94143 San Francisco, CA, USA; 2 University of California San Francisco Cancer Risk Program, 94115 San Francisco, CA, USA; 3 University of Michigan Medical School, 48109 Ann Arbor, MI, USA; 4 Department of Epidemiology and Biostatistics, University of California San Francisco, 94107 San Francisco, CA, USA; 5 Department of Obstetrics and Gynecology, University of California San Francisco, 94143 San Francisco, CA, USA; 6 Robert Wood Johnson Medical School, 08854 Piscataway, NJ, USA; 7 Department of Medicine, University of California San Francisco, 94143 San Francisco, CA, USA

**Keywords:** Family communication, Genetic testing, Hereditary breast and ovarian cancer syndrome, BRCA1, BRCA2

## Abstract

**Background:**

Individuals who carry deleterious BRCA mutations face significantly elevated risks of breast, ovarian, and other cancers. These individuals are also responsible for informing relatives of their increased risk for carrying the family BRCA mutation. Few interventions have been developed to facilitate this family communication process.

**Methods:**

We developed the Sharing Risk Information Tool (ShaRIT), a personalized educational intervention, to support BRCA carriers as they discuss BRCA positive results and their implications with relatives. We conducted a pilot study of 19 BRCA carriers identified through the University of California San Francisco Cancer Risk Program. Our study had two aims: 1) to assess the feasibility and acceptability of ShaRIT, and 2) describe characteristics associated with increased family communication and BRCA testing. Participants in our study were divided into two groups: those who had not received ShaRIT as part of their genetic counseling protocol (control group, n = 10) and those who received ShaRIT (n = 9).

**Results:**

All 9 women who received ShaRIT reported that it was a useful resource. Characteristics associated with increased sharing and testing included: female gender, degree of relationship, and frequency of communication. Increased pedigree knowledge showed a trend toward higher rates of sharing.

**Conclusions:**

Both participants and genetic counselors considered ShaRIT a well-received, comprehensive tool for disseminating individual risk information and clinical care guidelines to Hereditary Breast and Ovarian Cancer Syndrome families. Because of this, ShaRIT has been incorporated as standard of care at our institution. In the future we hope to evaluate the effects of ShaRIT on family communication and family testing in larger populations of BRCA positive families.

## Background

BRCA testing for women at high risk of Hereditary Breast and Ovarian Cancer (HBOC) Syndrome is recommended by multiple professional associations [1-3]. For women who test positive for a deleterious BRCA1 or BRCA2 mutation, the lifetime risk of breast cancer is up to 85% and the lifetime risk of ovarian cancer is up to 60% [4,5]. First-degree relatives of a BRCA carrier have a 1 in 2 chance of carrying the known family mutation, and second-degree relatives have a 1 in 4 chance.

One of the most clinically important ways to potentially decrease cancer incidence in HBOC is to target genetic testing towards families with known deleterious mutations. BRCA testing for a known family mutation costs a fraction of the price of full sequence BRCA testing, making it an extremely cost-effective way to identify at-risk BRCA carriers [6]. In HBOC families, knowledge of a relative's BRCA status is important to individualize recommendations for risk-reduction and screening [7]. Privacy laws preclude health care professionals from initiating contact with relatives, often leaving the "duty to warn" with the index carrier (the individual first identified as testing positive in a family) [8]. For these reasons, it is becoming increasingly important for BRCA carriers, and particularly for index carriers, to communicate genetic information with relatives.

Prior studies have examined sharing of BRCA results with at-risk relatives and their uptake of BRCA testing, as well as predictors of sharing and testing. Rates of sharing BRCA positive results with first and second-degree at-risk relatives in prior studies are quite high, ranging from 59%-86% [9-12]. BRCA carriers are more likely to share their results with female relatives and first-degree relatives, while infrequent contact and emotionally distant relationships have been identified as barriers to communication [10,13]. Despite high rates of communication, the uptake of genetic testing by at-risk family members remains significantly lower. Prior studies have found that as little as 36% of first- and second-degree relatives pursue BRCA testing, even if it is provided free of charge [9,14,15]. Data from our institution suggests that an index carrier's knowledge of hereditary breast and ovarian cancer, as well as her satisfaction with the decision to BRCA test are associated with her relatives' decision to undergo genetic testing [14].

When BRCA carriers inform relatives of their own results, they often also share information about their relative's risk of carrying a deleterious BRCA mutation. This risk communication process can be difficult, and important health information may be subject to non-communication, mis-communication, or mis-interpretation. Although BRCA carriers are educated about HBOC during genetic counseling and risk assessment, some studies show that several domains of knowledge remain low after genetic counseling, including understanding of gene transmission, risk probabilities among relatives, and how BRCA results can inform surveillance recommendations for relatives [15]. We hypothesize that a personalized educational program that supplements genetic counseling and focuses on sharing risk information with relatives is potentially an efficient and inexpensive way of increasing downstream genetic testing in at-risk relatives.

Few interventions to increase rates of genetic testing uptake in at-risk relatives have been developed [16]. Recent literature has called for a more tailored education of patients to improve family communication and testing [14]. The index BRCA tester is key to beginning this communication process. A recent review concluded with an appeal for the "development of an intervention to directly support people in talking to their relatives" [17]. One European study warned that communication can be a "children's whisper game, where many errors can occur in the transmission of DNA-test result information in families" [18]. It is therefore important to provide these index carriers with the appropriate tools to accurately communicate with family members regarding their potential risk of carrying a mutation.

To address these calls for an intervention and to study the process of sharing BRCA results with relatives, we developed a pilot program termed ShaRIT (Sharing Risk Information Tool). ShaRIT is a personalized informational tool that provides index patients with educational resources and support mechanisms, as well as personal risk assessment information for every at-risk first and second degree relative. ShaRIT strives to ease the "burden of the messenger" [19] and decrease the possibility of mis-communicating and mis-interpreting important medical information to their relatives [10,13,20-23].

This pilot study of the novel informational program ShaRIT aims to:

1. Test the acceptability and feasibility of ShaRIT from the perspectives of study participants and their genetic counselors.

2. Examine rates of sharing BRCA results and family testing in relatives, as well as predictors of increased sharing and testing, to inform future, larger studies.

## Methods

### Population

#### Recruitment

We recruited participants from the University of California San Francisco (UCSF) Cancer Risk Program, which provides genetic counseling and BRCA testing at the UCSF Helen Diller Family Comprehensive Cancer Center.

All women who received genetic counseling and tested positive for a germline BRCA1 or BRCA2 mutation at the Helen Diller Family Comprehensive Cancer Center between August 2009 and June 2010 were eligible for this study. Women who enrolled in the Institutional Review Board-approved UCSF Cancer Risk Program follow-up protocol [24] were contacted by phone or email in August and September 2010.

#### Eligibility criteria

Patients were excluded from participation if they met any of the following exclusion criteria: 1) had plans to move outside the United States within six months, 2) did not speak English at a 6^th ^grade level, 3) had any cognitive impairment (e.g. dementia), or 4) had a life expectancy of less than 12 months.

Inclusion criteria for relatives in the sharing and testing analyses were based on strict eligibility criteria designed by physician experts and genetic counselors through guidelines from the National Comprehensive Cancer Network [3] and genetic counseling guidelines set by the National Society of Genetic Counselors [2]. Relatives eligible for communication of BRCA positive results (sharing results) met the following criteria: no cognitive impairments, a life expectancy of at least 12 months, age ≥ 16 years old, 50% chance of carrying a mutation if a first-degree relative, and a 25% chance of carrying a mutation if a second-degree relative or cousin. To be eligible for testing for a known family mutation, relatives had to meet inclusion criteria for sharing and be at least 25 years old (the youngest age at which cancer screening is recommended if the relative tests positive). A panel of physician experts and genetic counselors convened to determine which relatives were eligible for sharing results and for family testing (based on age, medical history, and mutation risk).

**Figure 1:**
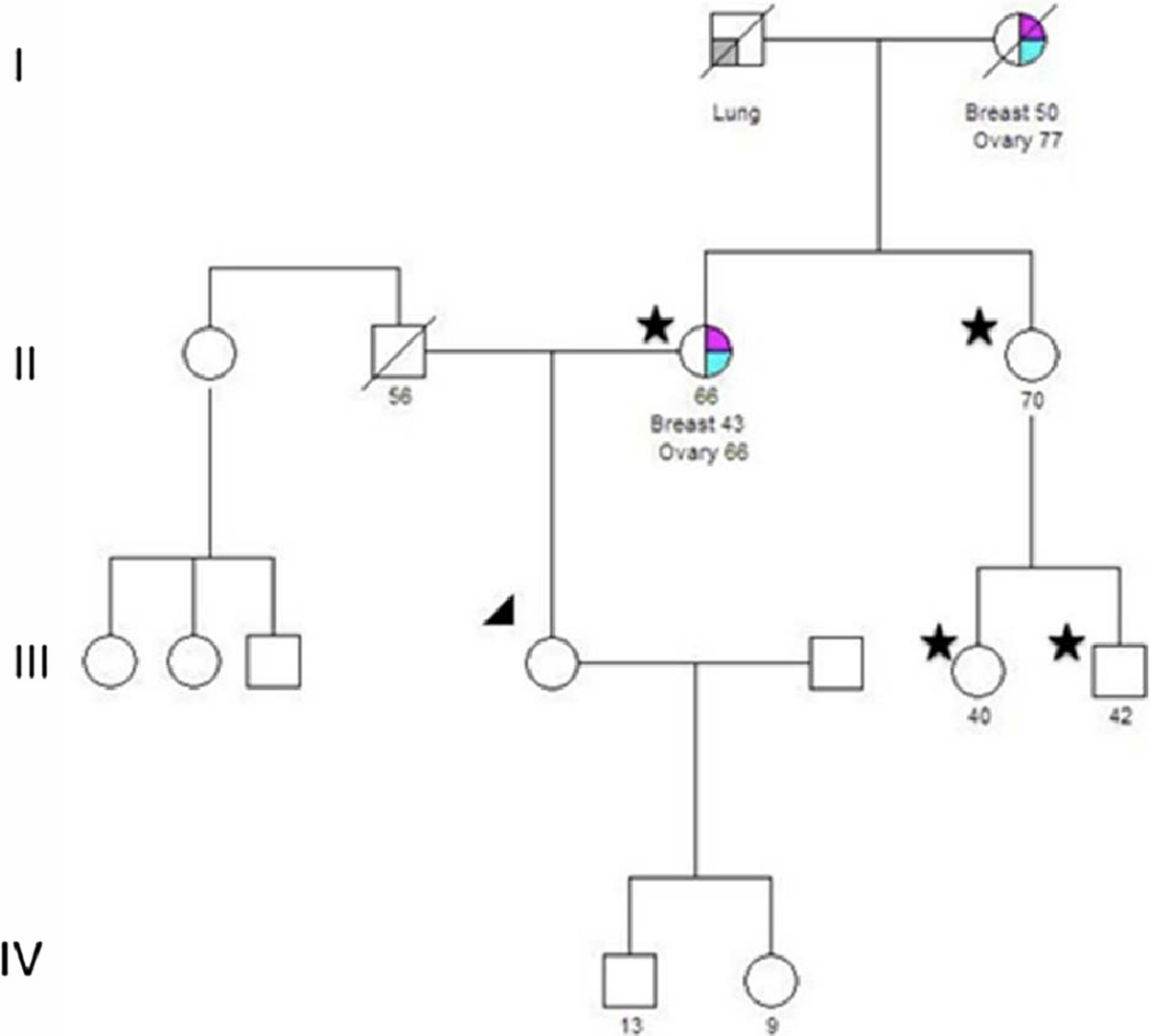
**Family pedigree of ShaRIT participant illustrating relative inclusion criteria**. The pedigree of the participant (arrow) shows a strong maternal family history of breast and ovarian cancer. Genetic counseling recommendations encourage testing of eligible relatives on the patient's maternal side. Stars denote the participant's mother, maternal aunt, and two maternal cousins, who are all eligible for sharing results and for family testing. The participant's daughter and son are too young to be included in sharing results or family testing analyses, as they are 13 and 9 years old.

**Figure 2:**
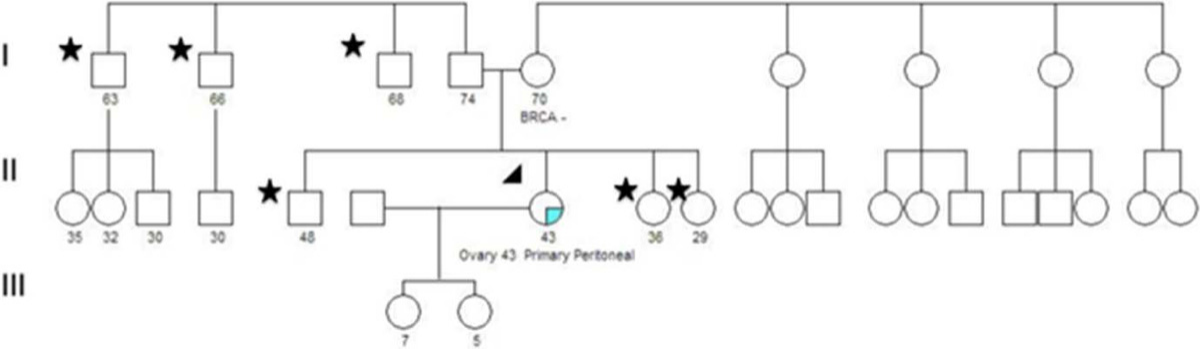
**Family pedigree of ShaRIT participant illustrating relative inclusion criteria**. The pedigree of the participant (arrow), who tested positive, does not show a strong family history of HBOC on either the maternal or paternal side. The participant's mother tested negative for the known family mutation, so it was likely inherited from the participant's father. The participant's brother, two sisters (first-degree relatives), and paternal uncles represent first and second-degree relatives eligible for sharing results and family testing (stars). The participant's two daughters are too young to be included in sharing results or family testing analyses, as they are 7 and 5 years old.

Figures 1 and 2 show family pedigrees of two ShaRIT participants to illustrate how study inclusion criteria were applied to relatives for sharing results and for family testing.

#### Control group

Before ShaRIT was introduced at the UCSF Cancer Risk Program, the following standard of care was practiced at the in-person genetic counseling visit to disclose BRCA results. After verbally informing patients of their BRCA positive status, they are given a single page lab report of their BRCA results. At this in-person visit, the implications of these results are discussed as they relate to the patient and to the patient's relatives. A longer personalized medical report (typically 3-4 pages) is sent to the patient by mail that describes and reviews the genetic testing process and the implications of the patient's BRCA test results. BRCA carriers are therefore informed in person and by mail of the importance of sharing genetic test results with family members. A single page "family letter" is provided to patients who express an interest in receiving help with communicating their BRCA results to relatives.

Patients who participated in the control group of this study tested positive between August 1, 2009 and February 1, 2010, signed an IRB-approved informed consent, and participated in the study phone survey.

#### ShaRIT group

The Sharing Risk Information Tool (ShaRIT), described in detail below, was incorporated into clinical practice at the UCSF Cancer Risk Program in February 2010. Patients who tested positive between February 1, 2010 and June 30, 2010, signed an IRB-approved informed consent, and participated in the study phone survey were included in the intervention, or ShaRIT group.

Table 1 describes the resources provided to participants in both the control and the ShaRIT groups as part of the BRCA results disclosure process.

**Table 1:** Resources provided to participants during results visit with genetic counselors

Items	Control	ShaRIT
**Personalized Medical Report**	Mailed after results visit	X
**Family Pedigree**	X	X
**BRCA mutation report from Myriad Genetics**	X	X
**Personalized Recommendations for Cancer Surveillance and Prevention**		X
**Information for Family Members:**		X
*Letter to family member notifying him/her of BRCA mutation identified in relative	Available to patient after results visit if requested	X
*FAQ for family members addressing cancer risk, cost of testing, insurance issues		X
*Contact information for genetic counselor(s) nearest to eligible family members		X
**General Information and Resources (Support group brochures, contact information for therapists with expertise in HBOC, general information on HBOC)**	X	X
**Files on CD-ROM**		X

#### Sharing risk information tool (ShaRIT)

ShaRIT is an educational tool of genetic information and family resources organized in a binder given to BRCA carriers during the in-person results disclosure visit with a genetic counselor. Each ShaRIT binder includes:

- The patient's personalized medical report, which is typically 3-4 pages

- Family pedigree

- BRCA mutation report from Myriad Genetics

- Personalized recommendations for surveillance and prevention

- Letter to family member notifying him/her of BRCA mutation identified in relative

- Fact sheet addressing frequently asked questions regarding cancer risk, costs of genetic testing, and insurance issues regarding genetic testing

- Contact information for genetic counselors specific to each eligible relative based on their geographic location. The National Society of Genetic Counselors website (nsgc.org) was used both as a reference and to identify genetic counselors near each of the eligible relatives.

- Support websites and brochures [25,26]

In addition to print versions of these resources, we provided each BRCA carrier with a personalized CD containing electronic versions of each resource included in the binder. Each BRCA carrier was counseled to use print and electronic ShaRIT resources to assist with family communication and to help answer common questions from relatives. They were encouraged to disseminate these resources to relatives, and to contact their genetic counselor for assistance and support throughout this process.

These personalized and standardized binders were developed in coordination with the physicians and genetic counselors at the UCSF Cancer Risk Program. These binders were used to guide and standardize the genetic counseling process, particularly as it relates to discussing family communication of BRCA results as well as BRCA testing of relatives.

### Survey instrument

We developed and pilot tested a semi-structured interviewer-administered survey, which lasted 15-30 minutes, depending on the number of relatives in each family. To allow time for sharing BRCA results with relatives, we waited at least 2 months after BRCA positive results disclosure before contacting the BRCA carrier (range 2-10 months post disclosure). To confirm family history and structure, we reviewed each participant's pedigree on file with the Cancer Risk Program before administering the phone survey. In addition to demographic data, the four content domains of the survey were:

1. Pedigree knowledge.

2. Family communication, sharing results, family testing.

3. Satisfaction with decision to BRCA test.

4. Acceptability of intervention.

### Feasibility evaluation

In addition to assessing acceptability of ShaRIT from the participant's perspective, we also assessed its feasibility from the genetic counselor's perspective. We surveyed all cancer risk genetic counselors within the UCSF Cancer Risk Program using an electronic survey conducted in November 2010.

### Measurements

#### Pedigree knowledge

Our knowledge construct consisted of six questions on the degree of relationship and heritability probabilities of the BRCA mutation for first-degree and second-degree relatives. We asked participants the following questions:

1) How would you define a first-degree relative?

2) Can you give me an example of one of your first-degree relatives?

3) What is the chance that a first-degree relative shares your BRCA mutation?

4) How would you define a second-degree relative?

5) Can you give me an example of one of your second-degree relatives?

6) What is the chance that a second-degree relative shares your BRCA mutation?

#### Family communication, sharing results, and family testing

The survey queried participants' reports of their frequency of communication with each eligible relative using a six-point scale (More than once a week, once a week, once a month, 1-6 times per year, once a year, and less than once a year), whether or not they shared their test results with each eligible relative, and whether or not, to their knowledge, each relative had pursued genetic testing for the known family mutation. If participants had shared their test results, we asked them how they disclosed the results, and whether they informed the relative directly of their results or if someone else had. If participants reported that their relative had pursued BRCA testing, we asked them if their relative had tested before them and how long ago they BRCA tested.

#### Satisfaction with the decision to BRCA test

We used a validated seven-question satisfaction with decision scale [27] to query the decision to undergo genetic testing for cancer risk. These questions assessed the following: feeling adequately informed about options, making a decision consistent with personal values, having adequate input in the decision, and having a positive effect on the participant's family. All questions used the same five-point Likert scale for responses.

#### Acceptability of intervention

We asked participants in the control group if they saved copies of their BRCA mutation report and personalized medical report. If they responded "yes" to either of these, they were asked if they shared these materials with their relatives. They were also asked if any relatives asked questions that they weren't comfortable answering. To assess for acceptability of ShaRIT components not previously distributed to BRCA carriers, we asked control group participants if they thought a single-page Frequently Asked Questions sheet, a list of relatives that should consider BRCA testing, or receiving information from their genetic counselor in an electronic format would have been helpful.

We asked participants in the ShaRIT group if they remembered receiving a binder at their results visit, and if so, whether they saved it. We asked if they shared any of the information in the binder with their relatives, and which materials they used and found most helpful. ShaRIT participants were also asked if any relatives asked questions that they weren't comfortable answering. We queried ShaRIT participants about aspects of the binder that were not helpful and about additional materials that should be included in future binders. Finally, we asked about the overall utility of ShaRIT, including the binder and the accompanying genetic counseling sessions.

The feasibility survey conducted among the four genetic counselors at the Cancer Risk Program who counsel patients at risk for HBOC asked the following questions:

1) How long does it take to prepare a ShaRIT binder for each patient?

2) Have you encountered any difficulties in preparing for ShaRIT?

3) Has ShaRIT increased/decreased your workload and time spent with patients? If so, how?

4) What is your overall satisfaction with ShaRIT, the final genetic counseling session, your communication with your patient, and the knowledge your patient gained regarding his/her test result?

5) List three things you like most about ShaRIT.

6) List three things you like least about ShaRIT.

### Study outcomes

The primary outcomes of this pilot study were the feasibility and acceptability of the ShaRIT intervention. These outcomes were assessed by interviewing genetic counselors and participants, respectively, using the questions described above in the Measurements section.

The secondary, quantitative outcomes of this study were 1) participants' report of sharing BRCA results with eligible family members and 2) participants' report of relatives receiving BRCA testing. Although it is possible that reports of BRCA testing could differ if participants or their relatives are queried, our consent process did not allow us to directly contact relatives. This approach has been followed in prior studies of our study outcomes [11,21].

### Data analysis

Descriptive and comparative statistics were used to characterize participants in terms of demographics, self-reported annual income, pedigree knowledge, satisfaction with decision to BRCA test, and mean number of relatives. We examined potential differences between control and intervention populations using *t *tests and *χ*2 tests, where applicable.

For analyses regarding the feasibility and acceptability of ShaRIT, we used quantitative and qualitative methods to record responses from the participant and genetic counselor surveys. We tallied these responses and also analyzed them for common themes.

For analyses regarding the communication of BRCA results to relatives, we classified responses by first-degree relatives, second-degree relatives, and cousins. We then compared sharing rates by these classifications. We followed a similar protocol for analyses of family testing. To measure reports of sharing test results with relatives and of relatives receiving genetic testing, we used a two-step process. First, we calculated the proportion of eligible relatives for each outcome by participant. Then, we measured frequencies of sharing and testing for each type of relative.

We analyzed the following characteristics that have been shown in prior studies to be associated with sharing and family testing: gender, type of relative, and frequency of communication. We also analyzed a novel measure, pedigree knowledge (as described above), for its association with sharing and family testing. Data analysis was done with Stata 12.0 statistical software (STATA Corp).

## Results

### Study population

During the eligibility period for this time-series analysis, we identified 37 women who met our inclusion criteria. Twenty-five of these were informed of their BRCA positive results between August 2009 and January 2010 (time period for the control group). Twelve were informed of their BRCA positive results between February 2010 and June 2010 (time period for the ShaRIT group). Eleven of the twenty-five women eligible for the control group participated in the phone survey (44%). Two of the women interviewed from the control group were cousins. Survey responses from the second woman interviewed from that family were excluded from analyses, to ensure that outcomes from this family were not counted twice. Nine of the twelve women eligible for the ShaRIT group participated in the phone survey (75%).

Study participants in the control and ShaRIT groups did not differ in their race, ethnicity, Ashkenazi Jewish ancestry, annual reported income, or satisfaction with decision to BRCA test (Table 2). Although not statistically significant, the control group was slightly older and had a wider range of ages. Women in the ShaRIT group had slightly higher pedigree knowledge scores, although differences in scores between groups were not statistically significant.

**Table 2:** Characteristics of BRCA positive participants (n = 19)

	Control	ShaRIT	p*
	n = 10	n = 9	
**Age**			
Mean	49 (± 8)	40 (± 6)	0.06
Range	26-63	33-49	
**Race/Ethnicity**			0.47
Caucasian	7 (70%)	7 (78%)	
Hispanic	2 (20%)	0 (0)	
African American	0 (0)	1 (11%)	
South Asian/Indian	0 (0)	1 (11%)	
Asian/Pacific Islander	1 (10%)	0 (0)	
**Ashkenazi Jewish**	2 (20%)	1 (11%)	1.00
**Annual Reported Income^a^**			1.00
≤ $100,000	2 (22%)	2 (22%)	
$100,001-$500,000	7 (78%)	7 (78%)	

**Pedigree Knowledge (scale 0-6)**	4.9 (± 1.4)	5.3 (± 1.3)	0.49
**Average Score**	4.3 (± 0.16)	4.5 (± 0.11)	0.23
**Satisfaction with Decision (scale 0-5)**			

* Fisher's exact test used for cell sizes ≤ 2 or percentages less than 20%

a. One participant in the control group declined to report her annual income

### Acceptability and feasibility of ShaRIT

#### Participant perspectives

In the control group, 70% of participants reported that additional information and resources regarding sharing results and family testing would have been useful. One control participant reported, "a Frequently Asked Questions (FAQ) sheet would have been helpful because there were a lot of questions I couldn't answer" from her relatives. Another suggested electronic versions of family letters and resources, as "it is much easier to talk about over email than over the phone." She also said she would have liked additional information regarding the "implications of (a) positive result" and "statistics on developing breast or ovarian cancer". 40% of the control group mentioned that electronic resources would have been useful, and 20% of the control group would have liked additional information on how a positive test result affects family planning for themselves and their relatives. One control group participant requested contact information of genetic counselors in other areas for her relatives.

In the ShaRIT group, 100% of participants reported that they saved their ShaRIT binder and they felt it was a useful resource. 44% reported that they gave or plan to give ShaRIT resources to their relatives, and 22% reported that additional information (more resources or handouts) would have been helpful to include in ShaRIT. For example, one ShaRIT participant would have liked a Spanish version of the FAQ sheet for her Spanish-speaking relatives. Only one ShaRIT participant felt the binder itself wasn't necessary. Three ShaRIT participants (33%) preferred the electronic CD-ROM version to the paper materials.

ShaRIT participants were asked open-ended questions about particularly useful components of the binder. One felt the binder was a "good means to give a package of information with options, results, recommended doctors, referrals, and the family tree" to relatives. Another reported, "I'm a visual person, and until I sit down and read it I don't get it reinforced. It was nice to have it down in writing." Yet another said that the mutation report was useful and that "it was good to have written information to give to my cousin about screening."

#### Genetic counselor perspectives

Genetic counselors at the UCSF Cancer Risk Program, who aided in the development of ShaRIT, have found the binder, accompanied by genetic counseling, to be both feasible and useful in clinically guiding high-risk patients through understanding the implications of positive results for them and their relatives. All genetic counselors queried reported that ShaRIT provides structure to their sessions. These counselors also all reported that ShaRIT allows results disclosure sessions to have a dual focus--on implications for the individual and for the family. One counselor said, "it (compiling ShaRIT materials) does increase my workload by about 30 minutes per patient, but it is worth it because I feel more organized and the patients seem to really appreciate the resources." When asked about her overall satisfaction with her communication with patients, the same counselor said, "It allows patients to be more focused on their feelings because they know that all the information they need is right in front of them."

### Sharing and testing in relatives

In total, there were 207 relatives eligible for sharing and 198 of those were considered eligible for testing by our criteria outlined above. Of the relatives eligible for sharing, 59 were first-degree relatives (37 in the control group, 22 in the intervention group), 57 were second-degree relatives (33 in the control group, 24 in the intervention group), and 91 were cousins (64 in the control group, 27 in the intervention group). Of the relatives eligible for testing, 54 were first-degree relatives (36 in the control group, 18 in the intervention group), 53 were second-degree relatives (33 in the control group, 20 in the intervention group), and 91 were cousins (64 in the control group, 27 in the intervention group). The median number and range of first-degree relatives, second-degree relatives, and cousins in both groups are shown in Table 3 as well as the proportion of relatives told and tested by participant.

**Table 3:** Characteristics and study outcomes of relatives by participant^a^

	Control (n = 10)	ShaRIT (n = 9)	p
**Median Number (Range) of:**			
First-Degree Relatives	3.0 (0-8)	2.0 (1-7)	N/A
Second-Degree Relatives	2.5 (0-9)	2.0 (1-6)	
Cousins	3.5 (0-27)	2.0 (1-11)	

**Shared Results with all Eligible Relatives**			
First-Degree Relatives	88%	90%	1.00
Second-Degree Relatives	38%	75%	0.32
Cousins	40%	63%	0.86
**All Eligible Relatives Tested**			
First-Degree Relatives	25%	20%	1.00
Second-Degree Relatives	67%	14%	0.10
Cousins	0%	0%	N/A

a. Proportion of relatives with whom results shared or who tested by participant. If participant did not know whether or not BRCA results were shared with that relative, or whether or not that relative tested, then fields were left blank. Fisher's exact test was used to compare sharing and testing outcomes between groups

Sharing rates for all first-degree relatives were over 95% overall; only two participants, one each in the control and ShaRIT groups reported not sharing their results with all of their first-degree relatives. Only 38% of control group participants told all of their second-degree relatives about their results, while 75% did so in the ShaRIT group. Sharing rates were similar for cousins across both groups.

Testing rates in eligible relatives were generally lower than sharing rates and displayed a similar decrease from first-degree relatives to second-degree relatives by participant group, except for second-degree relatives of control group participants. 67% of control group participants reported that all their eligible second-degree relatives tested, with only 14% of participants in the ShaRIT group reporting that all of their second-degree relatives had tested. No participants reported that all of their eligible cousins had tested. Among those participants aware of testing outcomes in their cousins, testing rates ranged from 0-71% in the control group and 0-50% in the ShaRIT group.

Table 3 shows the median number and range of first-degree relatives, second-degree relatives, and cousins for participants in the control and ShaRIT groups. For each participant, we calculated the percentage of each type of relative told about the participant's BRCA results. We analyzed relative uptake of BRCA testing in a similar manner.

**Figure 3:**
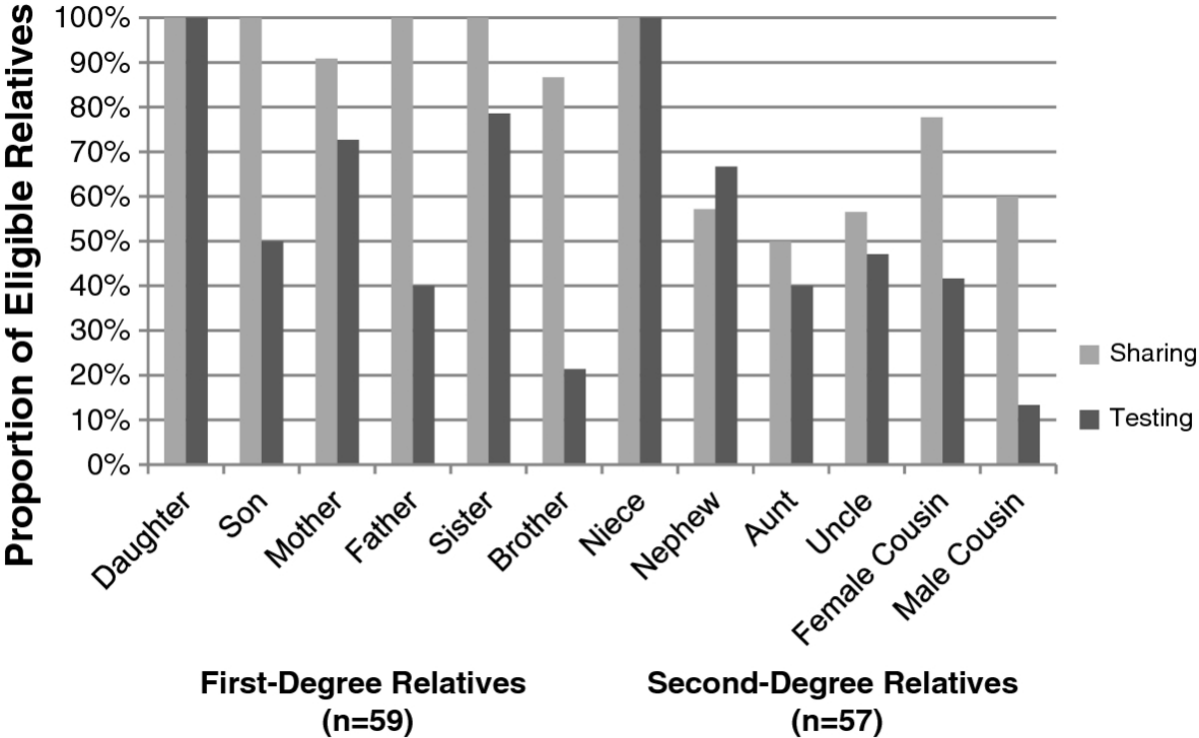
**Sharing and testing in eligible relatives based on relative gender and relationship to participant**.

We also examined patterns in sharing and testing based on relative characteristics for first-degree relatives, second-degree relatives, and cousins of participants. Female relatives were more often told about the participant's BRCA results, and were also more likely to undergo BRCA testing (Figure 3). Rates of sharing for first-degree relatives are very high, particularly when compared with sharing rates for all second-degree relatives except nieces. Sharing and testing outcomes for twelve different types of relatives are displayed in Figure 3, which was analyzed independent of intervention, using the combined control and ShaRIT groups.

**Figure 4:**
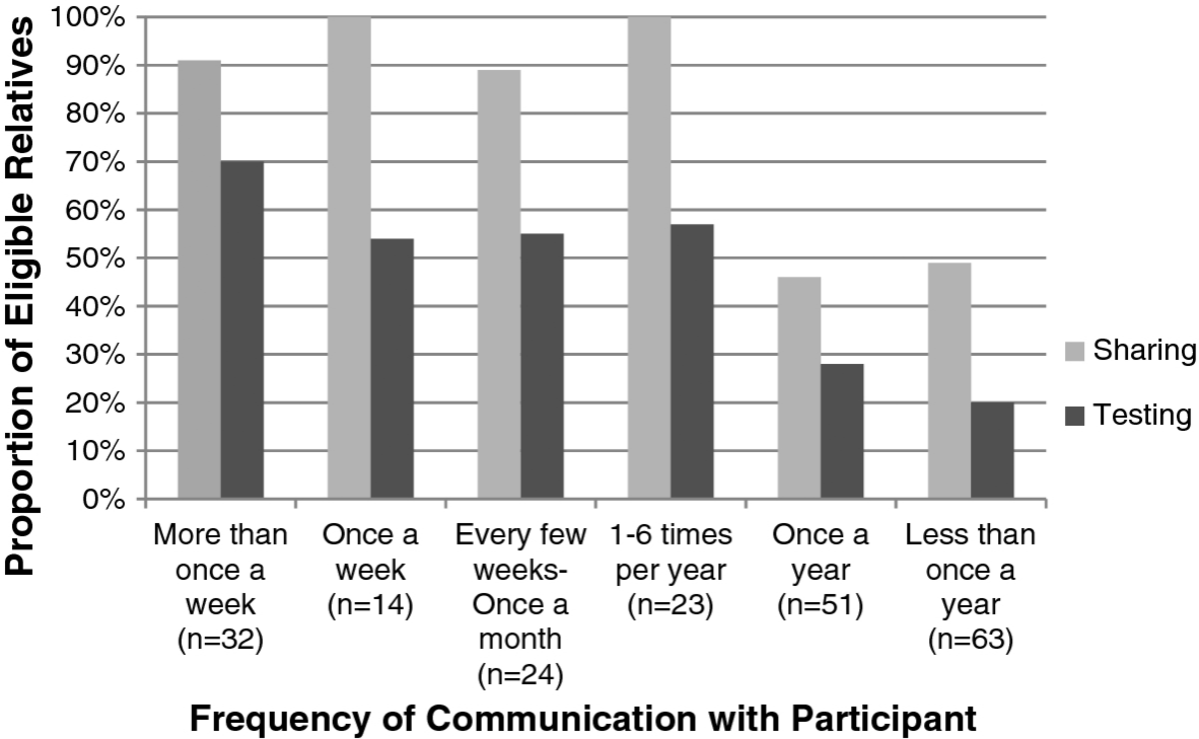
**Sharing and testing in eligible relatives based on frequency of communication with participant**.

Figure 4 shows the relationship between sharing and testing and frequency of communication between study participants (control and ShaRIT groups combined) and their relatives. In general, more frequent communication between participants and relatives was associated with more frequent sharing and testing.

**Figure 5:**
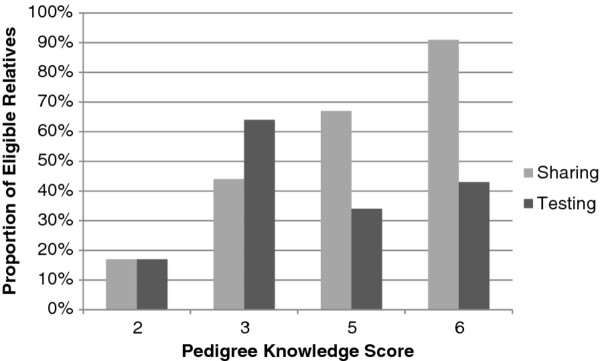
**Sharing and testing outcomes in all relatives based on participant's pedigree knowledge score**. Score was calculated as the number of correct responses out of six total questions. None of the participants scored 0, 1, or 4 on the pedigree knowledge questions.

Sharing and testing outcomes by participant based on pedigree knowledge score are shown in Figure 5. This demonstrates a trend toward increased communication with increasing levels of pedigree knowledge, but not necessarily increased testing with increasing pedigree score.

## Discussion

This pilot study of a personalized educational intervention, the Sharing Risk Information Tool (ShaRIT), demonstrates its acceptability and feasibility in clinical care for both patients and genetic counselors. We also used this pilot study to successfully test a phone survey instrument that measures rates of sharing results and testing relatives in families with a known BRCA mutation. Our results provide preliminary data that can be used to design larger studies and trials of interventions to improve risk communication in families with hereditary cancer syndromes. Additionally, our data confirms prior studies of characteristics associated with increased sharing and BRCA testing rates. Finally, we identified a new predictor associated in this pilot study with increased family communication: pedigree knowledge.

We believe the SharIT binder and accompanying genetic counseling sessions are feasible and acceptable ways to promote communication of BRCA test results to at-risk family members, as assessed by feedback received from both study participants and genetic counselors. The genetic counselors at the UCSF Cancer Risk Program felt that the additional 30 minutes required to compile the binder was worth their time as it better structured their counseling sessions with each patient and provided their patients with a resource to utilize after the final counseling session. All participants in the ShaRIT group were receptive to the binder as a reference to which they could refer at a later date and as a resource they could offer to their family members.

Our secondary aims to assess predictors of sharing and testing will better inform larger, future studies. Our results are similar to prior studies, which have demonstrated sharing rates of 59% to 86% in first- and second-degree relatives [9-12]. Our testing rates are also within the range of prior studies (36% of all eligible first- and second-degree relatives in one study; 57% in another) [9,15]. As with prior research, we found that first-degree relationships and female gender were associated with increased sharing and testing outcomes, particularly in the younger generations. In this pilot study, 100% of eligible daughters and nieces were reported to have received BRCA testing for the known family mutation.

Although cousins are third-degree relatives, we felt that it was important to assess their sharing and testing outcomes as well. In this pilot study, the number of at-risk cousins ranged from 0-27 per family. No families in either the ShaRIT or control group tested all eligible cousins (Table 3). Female cousins were more likely to BRCA test than their male counterparts (Figure 3). We consider this data on cousins a strength of our study, and a model for both counseling practice and future studies in this area.

We measured pedigree knowledge, the novel predictor of increased sharing rates that we identified, with six questions. Participants with higher pedigree knowledge reported higher rates of sharing BRCA results with relatives, but not necessarily higher rates of family genetic testing. This relationship implies that patients with greater knowledge may feel more comfortable communicating their BRCA results with relatives. With regard to testing, BRCA carriers can determine which relatives they choose to share their results with, but do not control their relative's decision to test for the known family mutation. Pedigree knowledge could prove to be an important variable that may be modified with targeted counseling and education. Further studies could examine whether increases in pedigree knowledge may result in improved sharing and family testing in larger populations of families with hereditary cancer syndromes.

This study is strengthened by the consideration of individual family structure--each participant's pedigree was studied before she was surveyed, and each eligible relative was individually assessed for sharing and testing outcomes. It is also strengthened by qualitative feedback from participants and from genetic counselors, which indicate both the feasibility and acceptability of ShaRIT.

We recognize several limitations of our study, which we have carefully considered in our interpretation of results. This was a pilot study of 19 families (10 control and 9 intervention), which made it difficult to sufficiently assess the independent predictive value of ShaRIT for family communication and testing outcomes. From these 19 families, however, we collected data on approximately 200 relatives eligible for sharing and family testing. Interestingly, participants in the control group had more second-degree relatives and cousins than the participants in the ShaRIT group, which may have further affected our ability to detect differences between the study groups. This pilot study was not powered to detect differences in sharing and family testing between the control and ShaRIT groups; rather, it served the purpose of demonstrating the feasibility and acceptability of ShaRIT. Our results provide estimates of the time, resources, and effect sizes needed to design a larger multi-site trial of ShaRIT.

This study design identified control and intervention groups based on the time of their genetic test result disclosure visit. Therefore, families in the control group had more time (up to eight additional months) to share results and to test relatives for the known family mutation. Prior studies have shown that sharing often occurs within a few weeks after genetic test result disclosure [28]. Testing of relatives, on the other hand, typically involves longer periods of time [14]. Because of the time series analysis of this study, it is possible that testing rates in the ShaRIT group would be higher if their follow-up time had been similar to that of the control group. We expect that longer follow-up of the ShaRIT group would identify higher testing rates for relatives. Future studies of ShaRIT and other similar interventions should consider designs to ensure similar follow-up times, including randomized controlled trials.

Because we recruited participants from a single site tertiary cancer center at a university hospital, it is unclear whether our results would generalize to other populations. The ethnic diversity of our population is reflected in that 26% of participants in this study were non-Caucasian. We plan to develop a Spanish version of ShaRIT and to test it in Hispanic populations throughout the United States. We are hopeful that we can perform future large multi-site studies of ShaRIT in diverse populations.

## Conclusions

To our knowledge, this pilot study is the first to examine the feasibility of a clinically- delivered, personalized educational intervention to improve communication and uptake of genetic testing in families with Hereditary Breast and Ovarian Cancer Syndrome. Our quantitative and qualitative results demonstrate the feasibility and acceptability of the Sharing Risk Information Tool (ShaRIT) for patients and for genetic counselors. Future research in this field should examine the impact of ShaRIT and other similar interventions in larger and more diverse populations in the setting of a multi-site randomized controlled trial.

## Competing interests

The authors declare that they have no competing interests.

## Authors' contributions

AK carried out data collection, patient recruitment, drafted the manuscript, and participated in study design. JF was responsible for data analysis, participated in the drafting of the manuscript, and manuscript revision. JC assisted with data analysis and revision of the manuscript. EC was responsible for creation of the study instrument and participated in revision of the manuscript. MB participated in study design and manuscript revision, as well as supervised AK, JF, and EC. All authors read and approved the final manuscript.
